# Induction chemotherapy and hepatic artery embolization followed by extended resection for locally advanced gallbladder cancer: a case report

**DOI:** 10.1186/s40792-023-01664-1

**Published:** 2023-05-15

**Authors:** Chisato Takagi, Michio Sato, Masato Tomita, Atsushi Sugita, Toshiki Tokuda, Koki Fujiwara, Nobutoshi Ando

**Affiliations:** Department of Surgery, International Goodwill Hospital, 1-28-1, Nishigaoka, Izumi-Ku, Yokohama-City, Kanagawa 245-0006 Japan

**Keywords:** Gallbladder cancer, Preoperative embolization, Conversion surgery, Hepatopancreatoduodenectomy

## Abstract

**Background:**

Surgical resection plays a critical role in the curative therapy of patients with gallbladder cancer. However, extended resection for locally advanced gallbladder cancer is a controversial procedure because of the high operative morbidity, mortality, and poor prognosis after surgery, without consensus of its suitability. Several reports have described preoperative treatment modalities to reduce the risk of mortality and morbidity and improve the curability of surgery for locally advanced GBCA. However, only a few well-designed studies have verified the benefits of these preoperative strategies.

**Case presentation:**

A 62-year-old male patient presented to our department with a gallbladder tumor detected on abdominal ultrasound during an annual medical checkup. Multi-phase enhanced CT revealed a gallbladder tumor with a maximum diameter of 34 mm, invading the right hepatic artery, pancreatic head, hepatic flexure of the colon, and first portion of the duodenum. We diagnosed gallbladder carcinoma as cT4 cN0 cM0 cStage IVA in the Union for International Cancer Control (UICC) classification 8th edition. After administration of 12 cycles of gemcitabine and cisplatin plus S-1 regimen, tumor shrinkage was observed on computed tomography, and elevated serum CA19-9 levels were reduced to normal limits. After preoperative hepatic artery embolization, we performed gallbladder bed resection with pancreaticoduodenectomy (minor hepatopancreatoduodenectomy) and combined resection of the right hepatic artery and hepatic flexure of the colon. Histological examination revealed no evidence of lymph node metastasis (ypT4 ypN0 ycM0 yp Stage IVA in the 8th edition of the UICC). The proximal bile duct and dissected margins were negative.

**Conclusions:**

The combination of induction chemotherapy and preoperative hepatic artery embolization, followed by minor hepatopancreatoduodenectomy and combined resection of the involved arteries and partial colon, could be a feasible treatment strategy for patients with locally advanced gallbladder cancer invading neighboring organs.

## Background

Surgical resection plays a critical role in the curative therapy of patients with gallbladder cancer (GBCA). However, some patients with GBCA are ineligible for curative surgery owing to delayed diagnosis from vague or non-specific symptoms, resulting in diagnosis at far advanced stages [1]. As GBCA frequently invades contiguous vessels and organs, some patients with advanced GBCA require extended surgery, such as hepatopancreatoduodenectomy (HPD) and/or combined resection of involved contiguous vessels and organs, to achieve R0 resection. However, such extended procedures have rarely been adopted because of their high postoperative morbidity, mortality, and poor long-term survival rate [2–4].

There have been several reports of conversion surgery for advanced GBCA following chemotherapy to improve resectability and long-term outcomes [5]. For the case of periampullary cancer that invades the right hepatic artery (RHA), pancreatoduodenectomy (PD) with combined resection of the RHA following preoperative arterial embolization was advocated to reduce operative mortality and morbidity due to ischemia of the bile duct and liver in several reports [6–8]. However, there were few well-designed studies verifying the efficacy of these preoperative strategies for patients with advanced GBCA.

Herein, we present a case of advanced GBCA, achieving R0 resection by gallbladder bed resection with PD (minor HPD) with combined resection of the right hepatic artery and hepatic flexure of the colon, preceded by systemic chemotherapy, and preoperative hepatic artery embolization.

## Case presentation

**Fig. 1:**
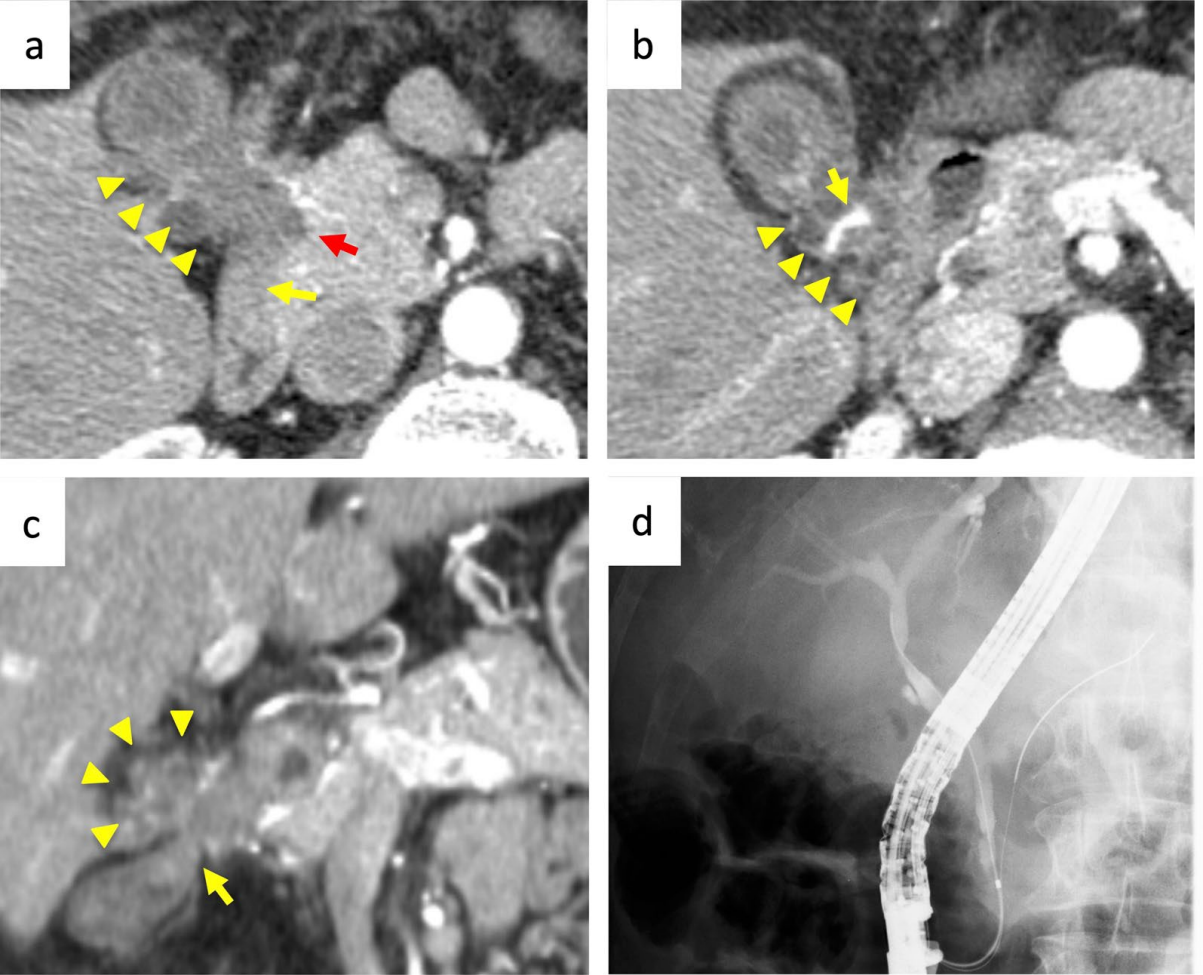
Initial radiological findings. **a** CT showed a gallbladder tumor (yellow arrowheads) located at the peritoneal side of gall bladder body. Infiltrations of the duodenum (yellow arrow) and the pancreas (red arrow) were also detected. **b** Axial image of 1.25 mm cranial side from (**a**). The tumor (yellow arrowheads) encased the RHA (yellow arrow). **c** CT coronal image showed that the tumor (yellow arrowheads) infiltrated into the hepatic flexure of the colon (yellow arrow). **d** Endoscopic retrograde cholangiography showed that a disruption of the cholecystic duct and the gall bladder was not contrasted, whereas the aliment of the bile duct was smooth. *CT* computed tomography; *RHA* right hepatic artery

A 62-year-old male patient presented to our department with a gallbladder tumor detected on abdominal ultrasound during an annual medical checkup. The serum carbohydrate antigen (CA) 19-9 was elevated (125.8 U/mL; normal range, ≤ 37 U/mL). Multi-phase enhanced computed tomography (CT) scans revealed a gallbladder tumor with a maximum diameter of 34 mm, located at the peritoneal side of the gallbladder body (Fig. 1a–c). CT and magnetic resonance imaging (MRI) showed that the tumor invaded the RHA, pancreatic head, hepatic flexure of the colon, and first portion of the duodenum, with suspicions of slight liver invasion. Furthermore, CT revealed variants of hepatic arteries, such as the accessory right hepatic artery (aRHA) arising from the superior mesenteric artery (SMA), replaced left hepatic artery (rLHA) arising from the left gastric artery, and middle hepatic artery (MHA) and RHA originating from the proper hepatic artery. rLHA, MHA, RHA, and aRHA supplied segments 2/3, 4, 5/7/8, and 6, respectively. The RHA and aRHA were encased by the tumor. There was no evidence of distant metastasis and bile duct dilation. We performed endoscopic retrograde cholangiopancreatography and collected bile for 3 days by endoscopic nasobiliary drainage for cytologic examination of malignancy, which proved to be class III (intermediate) (Fig. 1d). We performed endoscopic ultrasound-guided fine-needle aspiration of the tumor, revealing class III on cytologic examination. Although definitive evidence of malignancy was not obtained, we clinically diagnosed the gallbladder carcinoma as cT4 cN0 cM0 cStage IVA according to the Union for International Cancer Control (UICC) Classification 8th edition.

### Induction chemotherapy

We considered the necessity of induction chemotherapy in the present case, which invaded the neighboring organs, with the aim of downstaging. We administered a gemcitabine and cisplatin plus S-1 (GCS) regimen, which proved to provide a longer survival period than the gemcitabine and cisplatin regimen for patients with advanced biliary tract cancer in a phase III trial [9]. As a GCS regimen, the patient received gemcitabine and cisplatin administered intravenously at doses of 1000 mg/m^2^ and 25 mg/m^2^, respectively, on day 1, and oral S-1 was administered daily at a dose of 80 mg/m^2^ on days 1–7 every 2 weeks for a maximum of 12 cycles. The serum CA19-9 was temporarily elevated to 199.3 U/mL after two cycles and then reduced within the normal range of 28.3 U/mL. After 12 cycles, multi-phase enhanced CT revealed that the tumor diameter reduced from 34 to 27 mm (79% shrinkage rate), and no metastasis was detected. The patient did not experience more than grade 2 adverse effects, as defined by the Common Terminology Criteria for Adverse Events, version 5.0. Therefore, we planned to conduct conversion surgery.

### Preoperative arterial embolization

**Fig. 2:**
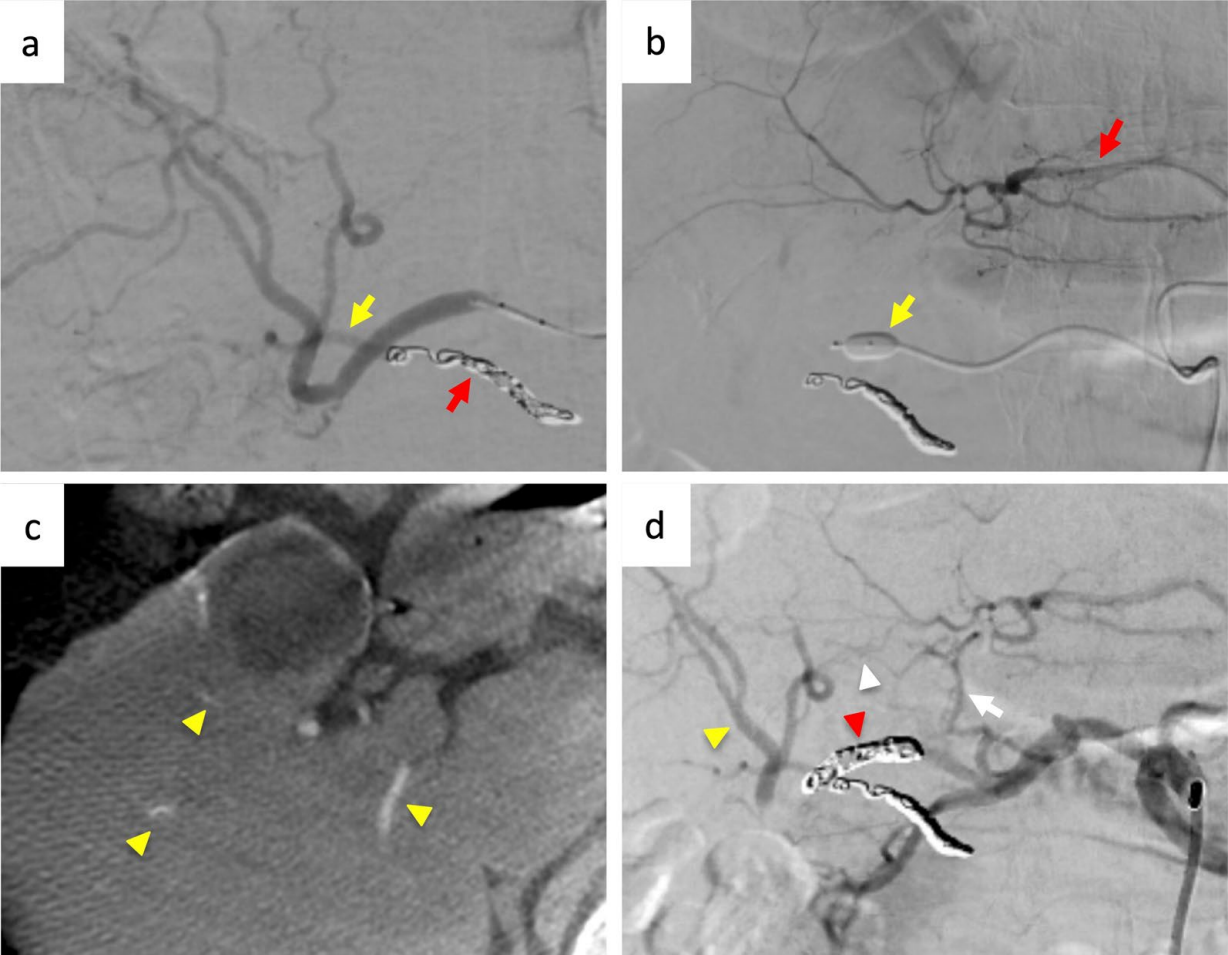
Preoperative angiography and hepatic artery embolization. **a** Selective angiography from the RHA after an embolization of aRHA (red arrow) showed the intrahepatic artery comprised of aRHA was compensated by RHA (yellow arrow). **b** Selective angiography of the rLHA after temporally occlusion of RHA using micro balloon catheter, which did not detect the collateral artery from LHA to RHA. **c** Cone-beam CT of (**b**) detected RHA (yellow arrowhead) fed by LHA. **d** Selective angiography of the celiac artery after an embolization of RHA (red arrowhead) detected LHA and the middle hepatic artery (white arrow) and collateral artery (white arrowhead) and RHA (yellow arrowhead). *CT* computed tomography, *RHA* right hepatic artery, *aRHA* accessory right hepatic artery, *rLHA* replaced left hepatic artery

**Fig. 3:**
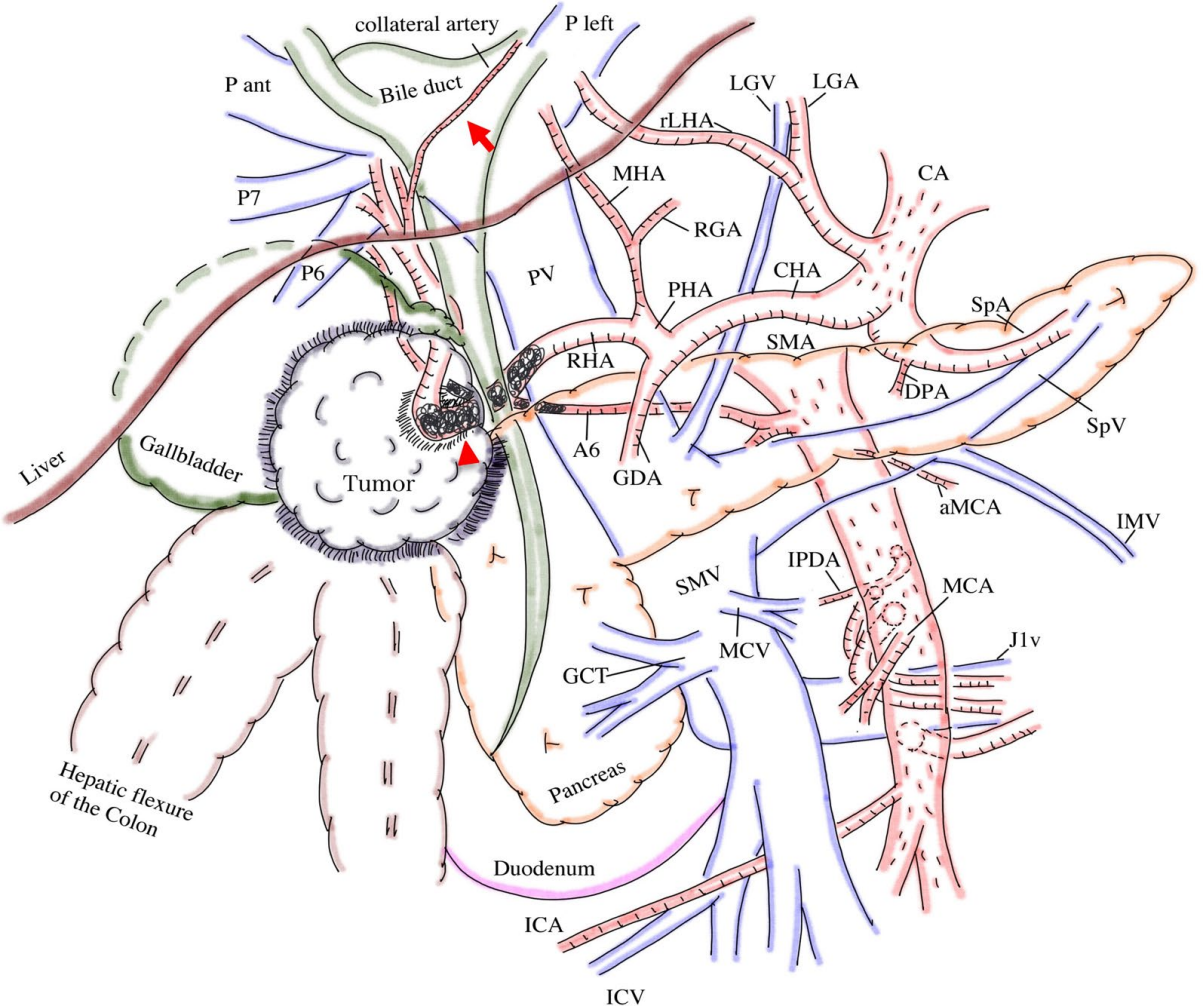
Scheme obtained by multi-phased CT after hepatic artery embolization. The RHA and the aRHA were occluded by coil (red arrowhead). A collateral artery from the left liver to the right liver was detected after embolization (red arrow). *aMCA* accessory middle colic artery, *aRHA* accessory right hepatic artery, *CA* celiac artery, *CHA* common hepatic artery, *DPA* dorsal pancreatic artery, *GCT* gastrocolic trunk, *GDA* gastroduodenal artery, *ICA* ileocecum artery, *ICV* ileocecum vein, *IPDA* inferior pancreaticoduodenal artery, *J1v* first jejunal vein, *LGA* left gastric artery, *LGV* left gastric vein, *MCV* middle colic vein, *MHA* middle hepatic artery, *MHV* middle hepatic vein, *PHA* proper hepatic artery, *P-ant* right anterior portal vein, *P-left* left portal vein, *PV* portal vein, *P7* portal vein of the segment 7, *RGA* right gastric artery, *RHA* right hepatic artery, *rLHA* replaced left hepatic artery, *SMA* superior mesenteric artery, *SMV* superior mesenteric vein, *SpA* splenic artery, *SpV* splenic vein

Given the necessity of combined aRHA and RHA resection for R0 resection, postoperative liver ischemia was considered. Thus, we performed preoperative arterial embolization by an interventional radiologist using angiography. First, we embolized the aRHA using a coil and ensured no ischemic area in the right lobe of the liver by selective angiography of the RHA (Fig. 2a). Then, we temporally occluded the RHA using a micro balloon catheter and simultaneously achieved selective angiography of the MHA and LHA to assure the collateral arteries from the left lobe to the right lobe of the liver (Fig. 2b). The collateral arteries were confirmed using cone-beam CT angiography (Fig. 2c). Finally, we embolized the RHA using a coil and confirmed the collateral arteries again by angiography of the celiac axis (Fig. 2d). After hepatic arterial embolization, elevated serum levels of aspartate aminotransferase and alanine aminotransferase were not observed. On day 7 after embolization, multi-phase enhanced CT showed that the blood flow of the intrahepatic artery in the whole liver was preserved. Moreover, CT revealed a pseudoaneurysm on the right femoral artery related to site of puncture. As the size of pseudoaneurysm was 2.6 cm in diameter and relatively small, we performed ultrasound-guided compression repair, and pseudoaneurysm disappeared and did not recur. Figure 3 shows a schematic of the vessel anatomy after arterial embolization.

### Operative procedure and postoperative short-term outcomes

**Fig. 4:**
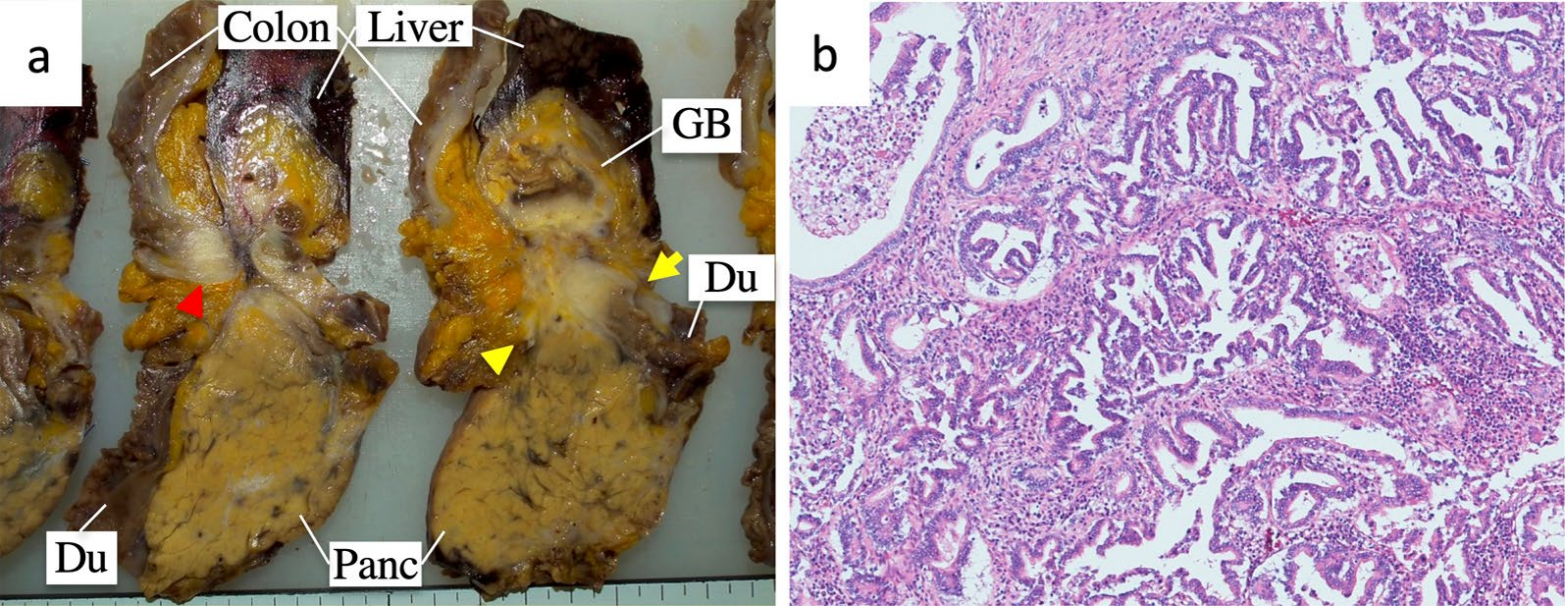
Macroscopic appearance and histological examination of the resected specimen. **a** Tumor arose from gallbladder and infiltrated the duodenum (yellow arrow), pancreas (yellow arrowhead), and colon (red arrowhead). **b** HE staining. Well-differentiated adenocarcinoma was observed in the gallbladder wall. *GB* gallbladder, *Du* duodenum, *Panc* pancreas, *HE* hematoxylin–eosin

Three weeks after embolization, we performed minor HPD and a modified Child’s method reconstruction with combined resection of the RHA, aRHA, and hepatic flexure of the colon. The operation time was 566 min, and the estimated amount of operative hemorrhage was 690 mL with no blood transfusion. We confirmed the blood flow of the artery, portal vein, and hepatic vein in the bilateral lobe of the liver using Doppler abdominal ultrasound during the operation and on postoperative days (POD) 1 and 2. On POD 8, multi-phase abdominal CT revealed intraabdominal fluid around the anastomosis of the pancreas and jejunum, and we performed abdominal ultrasound-guided drainage of the fluid collection which was considered owing to postoperative pancreatic fistula. In addition, the patient did not experience any other postoperative complications and was discharged on POD 24. The pathological findings were as follows: advanced gallbladder cancer ypT4 (invasion of the duodenum, pancreas, and hepatic flexure of the colon) ypN0 ycM0 ypStage IVA in the 8^th^ edition of the UICC Classification. After discharge, we administered S-1 as an adjuvant chemotherapy for 24 weeks. There was no evidence of recurrence on CT, 9 months after surgery (Fig. 4).

## Discussion

The GBCA presented in this case invaded the RHA with low likelihood of liver invasion as well as the pancreatic head, hepatic flexure of the colon, and first portion of the duodenum, defined as T4 in the UICC TNM Classification of Malignant Tumors 8^th^ edition. Radical resection, such as major HPD (two or more sections as an extension of liver resection), may be proposed to achieve R0 resection. However, according to the Japanese guidelines for biliary tract cancer, there is no general consensus on the benefits of major HPD for patients with GBCA because of the poor postoperative survival and high morbidity and mortality associated with this procedure [10, 11]. Thus, considering the surgical and oncological aspects of advanced GBCA in the present case, we considered that major HPD should be avoided, and a more appropriate treatment strategy should be explored, including preoperative treatment and less-invasive procedures, such as minor HPD.

In the present case, we planned conversion surgery after confirming a favorable response to induction chemotherapy. While R0 resection of GBCA is reported to contribute to long-term survival, R0 resection rates remain low in patients with GBCA involving neighboring organs, suggesting that careful case selection for resection is required [12]. The median survival time of resected T4 GBCA, similar to the present case, was reported to be about 12 months, which was an unsatisfactory result, although an improvement from the unresected T4 GBCA median survival time of 3 months [13]. A retrospective study evaluating preoperative chemotherapy in patients with locally advanced GBCA showed that improved survival rate was related to favorable responses to chemotherapy and R0 resection [4]. A multi-center retrospective study showed the survival benefits of conversion surgery followed by chemotherapy for patients with initially unresectable biliary tract cancer compared with chemotherapy alone [14]. Thus, as indication, induction chemotherapy could be more adequate for locally advanced GBCA than up-front surgery, and conversion surgery should be discussed after confirming a favorable response to induction chemotherapy. Moreover, as the selection of the regimen of induction chemotherapy, the regimen of induction chemotherapy was considered in terms of the response rate of regimens to obtain favorable response. In the present case, we believed that we could achieve minor HPD with combined resection of the RHA and partial colon at the time of initial diagnosis; however, we did not expect for up-front surgery to contribute to better outcomes because of the advanced tumor stage. Thus, we planned induction chemotherapy and conversion surgery after confirming a favorable response to induction chemotherapy. We administered the GCS regimen, which demonstrated a better response rate and survival benefit than the gemcitabine and cisplatin regimen in a multi-center, randomized phase III trial [9]. R0 resection was achieved after administering the GCS regimen for 12 cycles, which was designed as the maximum cycle in the clinical trial. The indication for conversion surgery was considered based on the following findings: reduction of serum CA19-9 level of 199.3 U/mL to the normal range and tumor size shrinkage on CT after chemotherapy. Although other findings of anatomical downstaging, including disappearance of vascular and other organ invasions, were not observed, R0 resection was expected to be achieved. Conversion surgery after a favorable response to systemic chemotherapy could be a reasonable treatment strategy and contribute to R0 resection in patients with locally advanced GBCA.

An appropriate operative procedure should be determined based on the possibility of performing R0 resection and minimizing the risk of postoperative complications. Given minor HPD with combined resection of the tumor invading RHA as an alternative procedure to major HPD, the risk of liver ischemia due to the division of the hepatic artery is not well-known. The liver is reported to have collateral arteries, such as the interlobar hepatic artery at the hepatic hilus, right inferior phrenic arteries, and right adrenal arteries [15, 16]. Thus, liver ischemia does not occur logically even after artery flow in one or more sections is interrupted. However, according to previous reports on PD in patients with replaced RHA (i.e., arising from the SMA), resection of the RHA could lead to temporal ischemia in the bile duct and the liver, entailing leakage of the hepaticojejunostomy and liver abscesses [17]. Vascular reconstruction of the resected RHA is considered as a valuable procedure for tumor resection required in PD necessary for combined resection of the RHA. Although it was reported to be technically challenging because of requiring meticulous technique using micro surgery, developments in hepatobiliary–pancreatic surgery made vascular anastomosis of the resected hepatic artery an established procedure [8, 18, 19]. As another valuable and alternative procedure, preoperative hepatic artery embolization followed by PD with combined resection of the replaced RHA without vascular reconstruction was advised as it reduces risks of postoperative complications [6–8]. Although the present patient did not have replaced RHA, preoperative radiological images showed that there were enough proximal and distal margins to embolize, ligate, and resect the RHA from the tumor. Both procedures could be applied for the present case, and we adopted PD with combined resection of the RHA after embolization, because our institution could achieve the procedure more easily. To the best of our knowledge, there have been no reports of minor HPD with combined resection of the RHA and partial colon after preoperative hepatic artery embolization in patients with advanced GBCA. Although the surgical procedure should be considered in well-selected GBCA cases, it may be worth considering in cases when the GBCA involves the RHA with minimal invasion of the liver.

The reported median waiting period between preoperative hepatic artery embolization and surgery was 8 days [6]. We initially planned surgery within 2 weeks after surgery in the present case. However, we delayed surgery because of treatment of a femoral artery pseudoaneurysm after angiography. Ultrasound-guided compression repair for treatment of a femoral artery pseudoaneurysm was reported to be non-invasive, efficient, safe, and cost-effective. A previous review article described that the treatment was effective for femoral artery pseudoaneurysm < 3 cm in diameter [20].

## Conclusions

Minor HPD with combined resection of the RHA and partial colon after induction chemotherapy and preoperative hepatic artery embolization could be a reasonable treatment strategy for well-selected patients with locally advanced GBCA. This treatment strategy could be considered when GBCA invades the RHA, duodenum, and pancreas and does not massively invade the liver. Moreover, patients are expected to tolerate the planned treatment well. For patients with advanced GBCA, a favorable response after systemic chemotherapy could be useful information that contributes to conversion surgery.

## Data Availability

The data supporting the conclusions of this article is included within the article.

## Abbreviations

ALT: Alanine aminotransferase
aRHA: Accessory right hepatic artery
AST: Aspartate aminotransferase
CA: Carbohydrate antigen
CT: Computed tomography
GBCA: Gallbladder cancer
GCS: Gemcitabine and cisplatin plus S-1
HPD: Hepatopancreatoduodenectomy
MHA: Middle hepatic artery
MRI: Magnetic resonance imaging
PD: Pancreatoduodenectomy
POD: Postoperative day
RHA: Right hepatic artery
rLHA: Left hepatic artery
SMA: Superior mesenteric artery
UICC: Union for International Cancer Control
